# Cutaneous complications to medical adhesives in left ventricular assist device patients: A retrospective cohort study

**DOI:** 10.1016/j.jdin.2024.11.007

**Published:** 2024-12-16

**Authors:** Brenda Umenita Akinniyi, Hala Rogers, Shawn Glocker, Farooq H. Sheikh, Michael A. Cardis

**Affiliations:** a Department of Dermatology, MedStar Washington Hospital Center/Georgetown University Hospital, Washington, District of Columbia; b MedStar Heart and Vascular Institute, Medstar Washington Hospital Center/Georgetown University Hospital, Washington, District of Columbia

**Keywords:** allergic contact dermatitis, cutaneous complications, general dermatology, left ventricular assist device, medical adhesives, medical dermatology, wound care

*To the Editor:* Left ventricular assist devices (LVADs) support advanced patients with heart failure as a bridge to transplantation or as a long-term solution for patients that are ineligible for a transplant. Managing LVAD patients involves securing the driveline and using occlusive dressings for wound care, typically changed weekly or more frequently posttransplant and in the setting of complications.^1^ Reports of allergic contact dermatitis (ACD) from wound dressings are increasing, yet data on ACD in LVAD patients remain limited. Therefore, we conducted a retrospective cohort study at our high-volume LVAD center to explore ACD incidence and associated cutaneous complications in LVAD-supported patients.

We reviewed charts of patients with heart failure with LVADs implanted between January 1, 2015, and June 1, 2023, who had the International Classification of Diseases, Tenth Revision, Clinical Modification (ICD-10-CM) code L23 for ACD. Data from Electronic Health Records included demographics, heart failure history, LVAD details, Fitzpatrick Skin Type, ACD diagnosis, dermatology involvement, and treatment. Patients with ACD unrelated to their LVAD were excluded. In total, 37 patient charts were included in our analysis. The majority were male (76%) with a mean age of 57.1 years. Most patients were Black or African American (86%) with nonischemic cardiomyopathy (92%), primarily treated with HeartMate 3 LVAD (87%). ACD at LVAD drive-line site occurred within 1 year of LVAD implantation in 57% of cases, with 57% of ACD diagnoses linked to concurrent infections. Consultations from dermatology services were received by 62% of ACD-diagnosed patients, averaging 2 follow-up appointments.

Although ACD from medical adhesives was documented in approximately 8% of our LVAD patients, adhesives are a common but not exclusive source.^2^ Our figures (Fig 1, *A* and *B*) included cases linked to both adhesives and other antiseptic substances, underscoring the complexity of identifying causative agents without patch testing. Patch testing confirmed sensitivity to ethyl cyanoacrylate in adhesives and fragrance mix in one patient (Fig 1, *A*). The known sources of ACD include Biopatch, Mediopore, Telfa, Chlorhexidine gluconate, ChloraPrep, and Tegaderm. Additionally, cyanoacrylate-based medical adhesives and acrylic-based dressings and adhesives, such as Tegaderm, have been implicated in the literature.^3^, ^4^, ^5^ The complexity of adhesive formulations, which often contain multiple potential allergens, complicates the identification and management of ACD. Given their availability and cost-effectiveness, we suggest a low threshold for using hypoallergenic bandages to prevent and manage ACD in LVAD patients, including potential prophylactic use in this population.

**Fig 1:**
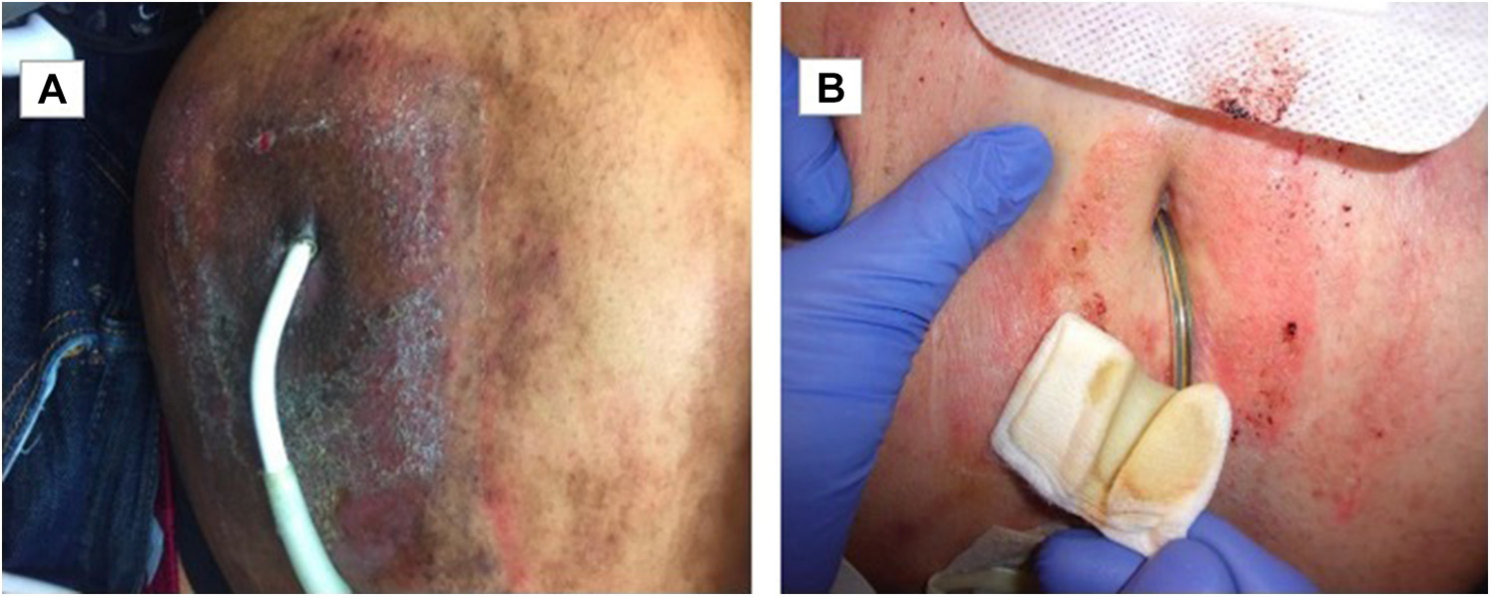
Allergic contact dermatitis at LVAD drivelines. **A,** A 49-year-old male, FST V, presenting with a DLES infection caused by *Pseudomonas aeruginosa*, occurring concurrently with chronic ACD. Patch testing confirmed sensitivity to ethyl cyanoacrylate in adhesives and fragrance mix. **B,** A 62-year-old male, FST II, presenting with ACD along the LVAD DLES in the setting of BIOPATCH Protective Disk with CHG adhesive usage. The patient was found to have a documented historical allergy to CHG. *ACD*, Allergic contact dermatitis; *CHG*, chlorhexidine gluconate; *DLES*, driveline exit site; *FST*, fitzpatrick skin type; *LVAD*, left ventricular assist device.

Our study contributes to LVAD patient care but acknowledges limitations. Distinguishing between ACD and irritant contact dermatitis using only ICD-10-CM code L23 was challenging, and nondermatologists made many diagnoses, increasing the risk of misclassification. The exact cause of ACD—whether due to adhesives or antiseptics—could not be determined. Concurrent infections may confound the diagnosis, with some cases potentially misclassified as ACD. Reliance on Electronic Health Records without confirmatory patch testing limits allergen identification, making it speculative to distinguish ACD from dermatitis caused by infection or other factors. Further research using standardized diagnostic protocols is needed to validate these findings.

## Conflicts of interest

Dr Sheikh is a consultant for Abbott. Author Akinniyi, Dr Rogers, Author Glocker, and Dr Cardis have no conflicts of interest to declare.
